# Fluid Flow and Mixing Induced by AC Continuous Electrowetting of Liquid Metal Droplet

**DOI:** 10.3390/mi8040119

**Published:** 2017-04-09

**Authors:** Qingming Hu, Yukun Ren, Weiyu Liu, Xiaoming Chen, Ye Tao, Hongyuan Jiang

**Affiliations:** 1 School of Mechatronics Engineering, Harbin Institute of Technology, West Da-zhi Street 92, Harbin 150001, Heilongjiang, China; qminghu@gmail.com (Q.H.); liuweiyu@chd.edu.cn (W.L.); xmchit@gmail.com (X.C.); tarahit@gmail.com (Y.T.); 2 School of Mechatronics Engineering, Qiqihar University, Wenhua Street 42, Qiqihar 161006, Heilongjiang, China; 3 State Key Laboratory of Robotics and System, Harbin Institute of Technology, West Da-zhi Street 92, Harbin 150001, Heilongjiang, China

**Keywords:** mixer, Marangoni chaotic advection, continuous electrowetting, liquid metal droplet

## Abstract

In this work, we proposed a novel design of a microfluidic mixer utilizing the amplified Marangoni chaotic advection induced by alternating current (AC) continuous electrowetting of a metal droplet situated in electrolyte solution, due to the linear and quadratic voltage-dependence of flow velocity at small or large voltages, respectively. Unlike previous researchers exploiting the unidirectional surface stress with direct current (DC) bias at droplet/medium interface for pumping of electrolytes where the resulting flow rate is linearly proportional to the field intensity, dominance of another kind of dipolar flow pattern caused by local Marangoni stress at the drop surface in a sufficiently intense AC electric field is demonstrated by both theoretical analysis and experimental observation, which exhibits a quadratic growth trend as a function of the applied voltage. The dipolar shear stress merely appears at larger voltages and greatly enhances the mixing performance by inducing chaotic advection between the neighboring laminar flow. The mixer design developed herein, on the basis of amplified Marangoni chaotic advection around a liquid metal droplet at larger AC voltages, has great potential for chemical reaction and microelectromechanical systems (MEMS) actuator applications because of generating high-throughput and excellent mixing performance at the same time.

## 1. Introduction

Mixing two or more streams within a confined microchannel is vital and challenging in the fields of chemical reaction [1,2,3], biomedical diagnostics [4,5], thermal management [6,7] and drug development [8]. Many approaches have been developed over the past few years to speed up the mixing, which can be mainly categorized into either active mixers or passive mixers in terms of driving mechanisms [9]. Typically, passive mixing increases the contact surface and contact time and disturbs the laminar flow mode between the neighbouring incoming electrolytes by incorporating intricate curved geometry or configuration of obstacles along the channel with an axial pressure-driven flow [10], such as zigzag channels [11,12], 3D serpentine structure [13,14], and embedded barriers [15,16]. Feng et al. presented four 3D micromixers with different arrays of crossing structures to accelerate diffusion process and investigated the effect of crossing sequences on the mixing efficiency [17]. However, the microfabrication process involved in this approach is sophisticated and considerably challenging. Alternatively, active microfluidic mixers utilize external energy to stir or agitate the fluid flow to promote the mixing performance, such as acoustic/ultrasonic [18,19], electro-hydrodynamic [20,21,22,23,24], magnetic [25,26], and laser techniques [27,28,29], etc. Among them, electrokinetic flow has been extensively employed in microfluidic devices to accomplish pumping and mixing, which refers to generating the flow stream instabilities on the surface of the micromachined electrodes activated by the electric field [30]. Oddy et al. explored electrokinetic instability occurring with sinusoidally oscillating and electroosmotic channel flows to rapidly stir two fluid streams at low Reynolds numbers in on-chip microfluidic systems [31]. Nevertheless, a very high driving voltage reaching up to 1–8 kV was required, which has the potential to generate bubbles on the microelectrode surface due to the electrolysis of working electrolyte. The alternating current (AC) electrical field was employed more frequently based on the virtue of its controllability of magnitude, phases and frequencies [32,33]—for example, the alternate current electrothermal (ACET) and alternate current electro-osmosis (ACEO). Sasaki et al. proposed AC electroosmotic flow originated from the interaction between the tangential electrical field and the double layer charge induced on the surface of a pair of coplanar meandering electrodes for mixing [34]. The electrothermally driven flow (ETF) arising from smeared structural polarization was exploited to create circulating flow on the surface of the electrode to transport small proteins by Sigurdson et al. [35]. However, the ACEO is merely produced for low-conductivity solution and low field frequency while the ACET effect could result in temperature rise in the microchannel and supernumerary Joule heating due to strong thermal-electrical coupling inside the fluid bulk, which sometimes associates with a detrimental effect to the suspending medium in the mixed streams. In addition, Li et al. explored the micro-mixer utilizing the induced charge electrokinetic flow around the electrically conducting particles and embedded triangular platinum hurdles in the microchannel generated by the interaction of the applied electric field with the induced dipolar electric double layers [36,37,38,39]. They also introduced the micro-valve using the net induced charge electro-osmotic flow motion around the mobile Janus particle [40,41].

Liquid metal eutectic alloy Galinstan (composed of 68.5% gallium, 21.5% indium and 10% tin by weight) has been extensively studied as a replacement for mercury in many applications due to its remarkable properties, including high electrical conductivity, high surface tension and nontoxicity [42], which makes them attractive for highly deformable and reconfigurable electronic devices [43]. A small Galinstan liquid metal droplet energized with a square wave signal was integrated into a liquid cooling system to facilitate heat dissipation through the cooling channel [7]. Tang et al. proposed a small-scale liquid metal pump enabled by electrowetting/deelectrowetting upon the application of a dynamic electric field to convert electrical energy into mechanical energy [44]. They also reported a soft liquid metal actuator utilizing continuous electrowetting effect to induce chaotic advection over the surface of a liquid metal droplet when activated by a sinusoidal signal [45]. The proposed microfluidic device demanded a copper droplet cap seat settled onto the polymethylmethacrylate (PMMA) substrate to locate the Galinstan droplet, hence a hole with height of 500 μm must be fabricated, which gives rise to high manufacturing costs and a tremendous fabrication process. Therefore, a satisfactory mixer with high throughput and convenient fabrication process is required urgently.

To simplify the fabrication process without moving mechanical parts, it seems reasonable to speculate on the usage of electrical wetting tension to induce chaotic advection over the surface of liquid metal droplet. In this work, we demonstrate a new and simple method to enhance the mixing efficiency through a continuous electrowetting base Galinstan liquid metal droplet mixer. A sinusoidal AC voltage signal was applied to induce inhomogeneity in surface tension of the metal droplet, which is a function of local double-layer voltage drop (native + induced), and the electrical Marangoni effect then ignites vortex flow on the edge region of the droplet by exerting hydrodynamic shear stress due to a surface gradient of the surface tension at the droplet/medium interface. The mixing process could be accomplished instantaneously and the mixing performance is excellent due to quadratic voltage-dependent growth trend of local bipolar Marangoni shear stress, in stark contrast to the linear law of unidirectional stress component that causes a pumping effect. In addition, the mixing performance can be improved by changing the magnitude and frequency of the applied electrical field, as well as the ion concentration of the electrolyte. Such a chaotic advection mixer triggered by continuous electrowetting behavior in the AC electric field based on Galinstan liquid metal droplet would be useful in a variety of applications including biomedical assays, drug development and heat transfer field.

## 2. Theory and Methods

### 2.1. Design and Fabrication of the Microfluidic Device

**Figure 1:**
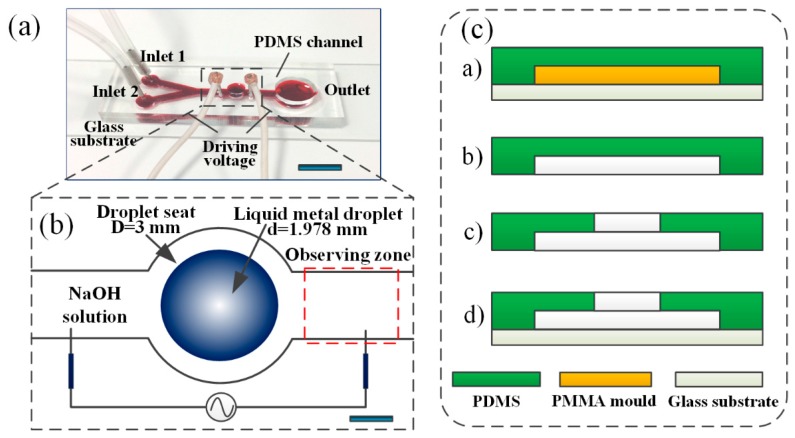
Design and fabrication process of the microfluidic mixer. (**a**) The schematic view of the liquid metal based microfluidic mixer. Scale bar, 6 mm; (**b**) the detailed mixer actuator. Scale bar, 500 μm; (**c**) fabrication processes of the microfluidic mixer including: (a) polydimethylsiloxane (PDMS) was poured and cured onto a glass slide; (b) the PDMS mixture was peeled off; (c) holes were punched; (d) PDMS channel was plasma bonding onto a glass slide.

As depicted in Figure 1a, the liquid metal based microfluidic device consists of a glass slide and a 5 mm thick polydimethylsiloxane (PDMS) containing a Y-shaped inlet channel, and a cylindrical chamber with a diameter of 3 mm was designed to locate the Galinstan liquid metal droplet with 1.978 mm in diameter. The channel width and depth are 1.2 mm and 1.3 mm, respectively. The electrodes are distributed on both sides of the cylindrical cap along the microchannel and has a distance of 6 mm. The detailed structure of the mixer can be illustrated in Figure 1b.

The PDMS layer was fabricated using a conventional soft-lithography procedure. The PMMA serving as chamber mold for the channel was prepared with laser cutter (K3020, Huitian, Dongguan, China), which was adhered to the glass substrate with glue. Subsequently, the PDMS (Sylgard 184, Dow Corning Inc., Midland, MI, USA) mixing with a curing agent (Sylgard 184 A & B, Dow Corning, Inc.) at a volume ratio of 10:1, was vacuumized for 30 min in a vacuum chamber and then poured onto the prepared PMMA master mold. After curing for 3 h at 80 °C in an oven, the PDMS mixture was peeled off, punched mechanically by biopsy punch (PSAM14, Acuderm, Fort Lauderdale, FL, USA), plasma treated with oxygen plasma (ZEPTO, Diener, Ebhausen, Germany), and then bonded onto a glass slide. Afterwards, the device was cured for 3 h at 80 °C [8]. The above fabrication process of the microchannel is shown in Figure 1c.

### 2.2. Theoretical Framework of AC Continuous Electrowetting

#### 2.2.1. Double Layer Voltage Drop Caused by the Native Surface Charge at the Droplet Surface

**Figure 2:**
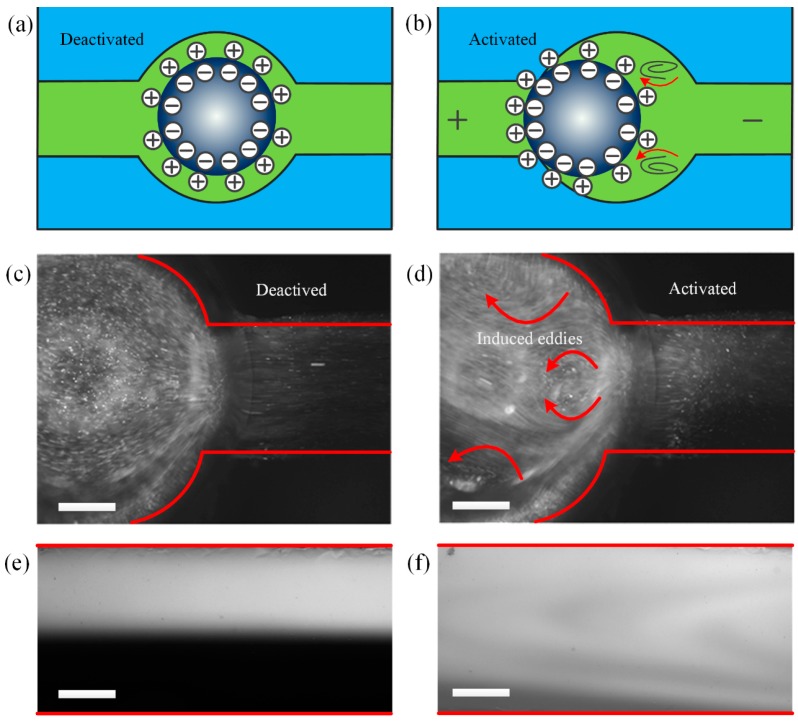
Working mechanism of the liquid metal droplet based high throughput microfluidic mixer. (**a**) Schematic of the electric charge distribution over the surface of the liquid metal droplet when immersed in NaOH solution; (**b**) schematic of the electric charge redistribution and the induced eddies over the surface of the liquid metal droplet along with the application of external electric field (note the fluid motion is caused by Marangoni effect but not Coulomb force within the diffuse double-layer); (**c**,**d**) microscopic optical image of fluorescent particle trajectories along the surface of the droplet observed from the top by the high speed camera before and after a sinusoidal wave of 6 Vpp and 100 Hz is activated between the two electrodes; (**e**) microscopic view of the flow pattern before the mixer is activated; (**f**) microscopic view of the flow pattern after the application of the electric field. The scale bar on each image is 500 μm.

When the Galinstan eutectic alloy liquid metal is immersed in a basic electrolyte, [Ga(OH)_4_]^−^ will be generated due to the chemical reaction between the gallium and the solution, causing an accumulation of oppositely charged ions on the surface of the liquid metal droplet, which results in the formation of electric double layer (EDL) in the diffuse layer, as illustrated in Figure 2a.

To the first order, native surface free charge density *q*_0_ is uniform at drop/electrolyte interface. Within Debye–Huckel limit and based on the Poisson–Nernst–Planck equation, the unipolar diffuse screening cloud $\rho_{0}=-q_{0}/\lambda_{d}e^{-r/\lambda_{d}}$ (where *ρ*_0_ is the native spatial charge density, *λ_d_* is the double layer thickness and *r* is the distance to the droplet centre) exponentially screens the bulk field $-\partial \phi /\partial r=q_{0}/\varepsilon e^{-r/\lambda_{d}}$ emitted by the surface charge *q*_0_, due to a balance between electrostatic attraction and surface ion diffusion, producing a fixed voltage drop $-\zeta_{0}=-q_{0}/C$ across the diffuse double layer of length scale $O\left(\lambda_{d}=\sqrt{D\varepsilon /\sigma }\right)$. Here, ϕ is the electrostatic potential, *ε*, *D* = 2 × 10^−9^ m^2^·s^−1^ and *σ* are the liquid permittivity, the ionic diffusivity and the conductivity of the suspending medium, respectively, *C* = *ε/λ_d_* is the double layer capacitance.

#### 2.2.2. Induced-Charge Electrokinetics

As an AC electric field is applied to the channel, bipolar ionic charges are attracted to the surface of conducting drop acting as a mobile floating electrode of inducing voltage $\phi_{drop}=\int_{\partial drop}\phi dS/\int_{\partial drop}dS$. Since ions are blocked from the ideally polarizable surface, at the outer edge of Debye layer, normal conduction current from the bulk should relay the displacement current flowing across the double-layer under the approximation of small induced zeta potential:

(1) $$\sigma n\cdot \nabla \tilde{\phi }=j\omega C\left(\tilde{\phi }-{\tilde{\phi }}_{drop}\right),$$

where ***n*** is the unit normal vector pointing from the droplet surface into the centre, ϕ denotes the electrostatic potential just outside the induced double-layer (IDL), and ϕ=Re(ϕ˜ejωt) with the capped variable representing the corresponding phasor for sinusoidal actuation and linear response. In direct current (DC) limit, the induced zeta potential $-{\tilde{\zeta }}_{i}=-\left({\tilde{\phi }}_{0}-\tilde{\phi }\right)∼-O\left(E_{0}x\right)$, x is the horizontal distance from the drop centre, i.e., $-E_{0}R<-{\tilde{\zeta }}_{i}<E_{0}R$ at the surface of droplet of radius *R*. The induced surface free charge density at the surface of floating drop ${\tilde{q}}_{i}=CE_{0}x$ is clearly dipolar and produces a bipolar conduction current, hence the induced screening cloud ${\tilde{\rho }}_{i}=-CE_{0}x/\lambda_{d}$ of a counter bipolar nature accumulated within the IDL tends to neutralize the dielectric system.

At frequencies larger than the reciprocal resistance-capacitance (RC) relaxation time $f_{RC}=\frac{\sigma \lambda_{d}}{2\pi \varepsilon R}$, the polarity of AC electric field changes so fast that accumulation of free charge within the diffuse double-layer is not allowed, resulting in ineffective fluid flow. As for a small pre-existing charge and applied voltage, the total voltage drop across the double-layer (external-internal) is given by the superimposition principle in electrochemistry:

(2) ΔV=(−ζ0)+(−ζi)=−q0/C+Re((ϕ˜−ϕ˜drop)ejωt).

#### 2.2.3. Fluidic Physics

Thermal dynamics predicts that the surface tension coefficient *γ* at the polarizable interface of a metal droplet is a function of the local double-layer voltage drop [44,46]:

(3) γ=γ0−12C(ΔV)2=γ0−12C(−q0/C+Re((ϕ˜−ϕ˜drop)ejωt))2,

where *γ*_0_ denotes the surface tension in the absence of an electric field. Neglecting the deformation of liquid droplet caused by normal Newtonian stress and the viscous effect at the side of metal drop, surface stress balance at the interface of two phase flow is given by an equilibrium between the tangential hydrodynamics shear stress and any possible external stress, as well as the normal pressure drop counter-balanced by the local surface tension on a curved surface [47]:

(4a) $$\mu \left(\nabla u+\nabla u^{T}\right)\cdot n\cdot t\cdot t=\nabla_{t}\gamma \left(\Delta V\right)=\left(\nabla \gamma -n\cdot \left(n\cdot \nabla \gamma \right)\right),$$

(4b) $$\Delta p\cdot n=\frac{2\gamma }{R}\cdot n,$$

where *μ* is the dynamics viscosity of the aqueous media, ***t*** is the unit tangential vector on the droplet surface, and Δ*p* is the pressure difference between the droplet and the surrounding medium. The viscous shear stress driving the ambient bulk flow comes from the surface gradient of the surface tension (Equation (4a)), which is dependent on the local double-layer voltage (Equation (3)) consisting of both a native one and an induced one (Equation (2)). The combination of Equations (1)–(4) describes the AC-electric-field-induced Marangoni boundary convection, which occurs only if an background electric field is supplied to render the double-layer charge (Equation (1)) and resulting surface tension non-uniformly distributed at the surface of liquid metal.

#### 2.2.4. Analytical Approximation at Low Frequency

To enable a better understanding of the continuous electrowetting phenomenon in an AC field, we present the analytical solution at low field frequency, assuming the metal droplet extends infinitely long along the *x*-direction:

(5a) $$\Delta V\left(x\right)=\left(-\zeta_{0}\right)+\left(-\zeta_{i}\right)=-q_{0}/C-E_{0}x\cos\left(\omega t\right),$$

(5b) $$\gamma \left(x\right)=\gamma_{0}-\frac{1}{2}C{\left(q_{0}/C+E_{0}x\cos\left(\omega t\right)\right)}^{2},$$

(5c) $$\frac{\partial \gamma }{\partial x}=-\left(q_{0}E_{0}\cos\left(\omega t\right)+E_{0}^{2}\cos^{2}\left(\omega t\right)Cx\right),$$

(5d) $$\tau \left(x,y\right)=-\left(q_{0}E_{0}\cos\left(\omega t\right)+E_{0}^{2}\cos^{2}\left(\omega t\right)Cx\right)\left(1-n_{x}^{2}\right)\vec{e_{x}}+\left(q_{0}E_{0}\cos\left(\omega t\right)+E_{0}^{2}\cos^{2}\left(\omega t\right)Cx\right)n_{x}n_{y}\vec{e_{y}}.$$

**Figure 3:**
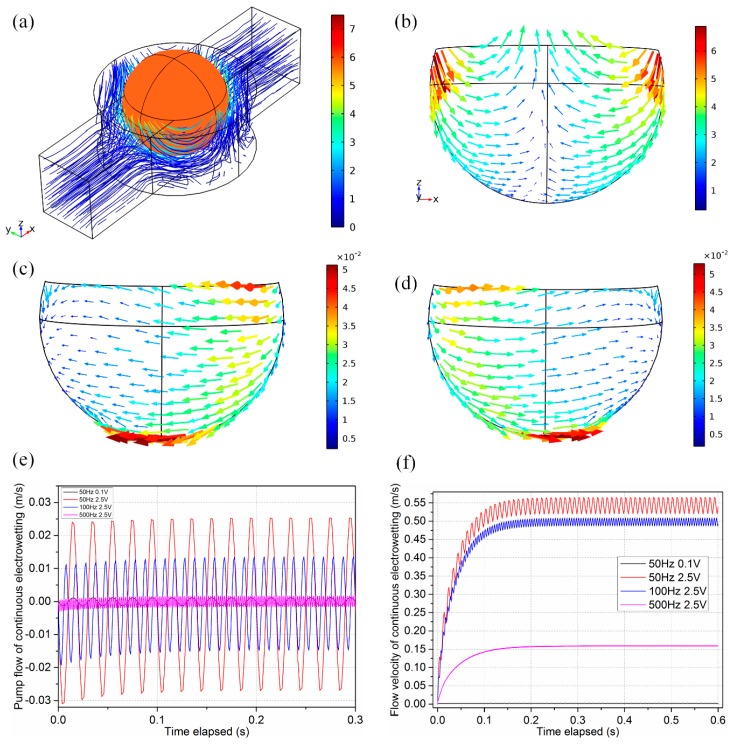
Theoretical quantification of fluid motion caused by metal droplet immersed in electrolyte solution in a background AC electric field. (**a**) Transient flow streamlines at an arbitrary time instant for the typical experimental condition *V*_0_ = 2.5 V, *f* = 50 Hz, *L* = 6 mm, *E*_0_ = 416.67 V/m, α ≈ 2.5; (**b**) the dipolar flow pattern at the droplet surface mainly caused by *τ_local_* for α ≈ 2.5 at *f* = 50 Hz and *E*_0_ = 416.67 V/m (2.5 V); (**c**,**d**) the unidirectional fluid motion induced by *τ_globe_* for α ≈ 0.1 at *f* = 50 Hz and *E*_0_ = 16.67 V/m (0.1 V) is always in the direction of the applied electric field; (**c**) within the first half cycle of AC field with **E**_0_ being along the negative *x*-direction, the fluid flow is in the negative direction as well; (**d**) within the second half cycle of AC field , both fluid motion and *E*_0_ are along the positive *x*-direction; (**e**) pump flow component *u_x_* and (**f**) total convective flow velocity *u* generated by AC continuous electrowetting under the condition of various voltage amplitude and field frequency.

Two transient shear stress and flow components can be well distinguished from one another:

(6a) $$\tau_{globe}=-q_{0}E_{0}\cos\left(\omega t\right),$$

(6b) $$\tau_{local}=-E_{0}^{2}Cx\cos^{2}\left(\omega t\right),$$

(6c) $$u_{globe}∼\chi \frac{-q_{0}E_{0}R}{\mu }\cos\left(\omega t\right)\;(\mathrm{with}\;\chi <1),$$

(6d) $$u_{local}∼\frac{-E_{0}^{2}RCx}{\mu }\left(\frac{1+\cos\left(2\omega t\right)}{2}\right),$$

where *τ_globe_* is a globally uniform shear stress acting on the drop surface, and causes a global pump effect along the longitudinal channel. Since *q*_0_ is commonly negative, with a DC bias, the pump flow $u_{globe}∼\frac{-q_{0}E_{0}R}{\mu }$ is in the direction of the applied electric field **E**_0_. However, in the absence of DC bias and under AC actuation, the net flow $u_{globe}∼\chi \frac{-q_{0}E_{0}R}{\mu }\cos\left(\omega t\right)$ (with χ<1) caused by *τ_globe_* alternates direction periodically as polarity of the applied field changes (Figure 3c,d), so a zero time-averaged pump effect may be engendered in AC (Figure 3e).

*τ_local_* denotes a local shear stress component, scaling linearly with the distance *x* to the sphere centre. The flow field $u_{local}∼\frac{-E_{0}^{2}RCx}{\mu }\left(\frac{1+\cos\left(2\omega t\right)}{2}\right)$, induced by *τ_local_*, fluctuates with time but does not reverse direction (Figure 3b). In addition, *u_local_* exhibits a pattern of dipolar shape at a half side of the metal droplet, and the flow velocity vector converges towards the symmetrical equator plane. Furthermore, it induces transient and chaotic convective vortices around the metal drop (Figure 3a) due to its unique bipolar flow streamline, which greatly accelerates the mixing process of parallel incoming analytes.

A nondimensional parameter *α* is defined to depict the ratio of the local shear stress to the global one:

(7) $$\alpha =\tau_{globe}/\tau_{local}∼\frac{E_{0}CR}{q_{0}}.$$

In our experiment, α ≈ 2.5 > 1, $\tau_{local}=-E_{0}^{2}Cx\cos^{2}\left(\omega t\right)$ dominates, so although the direction of net pump flow induced alternates with time (Figure 3e), the local vortex flow field on the drop surface may not (Figure 3b).

To make the fluid flow effective for convective mixing, the flow speed around the channel boundaries (where the flow becomes weakest) must exceed ten times the inlet flow velocity containing the incoming analyte:

(8) $$\langle u\rangle \approx \frac{E_{0}^{2}R^{2}C}{2\mu }\frac{R}{H}\geq 10u_{in}.$$

In our experiment, *E*_0_ = 417 V/m, *u_in_* = 100 μL/min, *H* = 1.3 mm, *C* = 0.6 F·m^−2^, so the theoretical minimum value of *R* should attain 65 μm. In practice, *R* = 989 μm is chosen to actuate sufficient strong chaotic advection for convective mixing.

### 2.3. Numerical Simulation

Direct numerical simulation is carried out on the basis of the theoretical framework outlined in Section 2.2, in order to comprehend the behavior of fluid flow in the presence of liquid metal induced by an AC electric field, using the COMSOL Multiphysics 5.0 Software Package (COMSOL AB, Stockholm, Sweden), which implements finite element analysis. The simulations are conducted in *frequency domain for electric field* while in a *fully transient manner for field-induced fluid motion*.

#### 2.3.1. Electrostatics

Assuming homogeneous ionic concentration in the bulk fluid, the electrostatic potential is given by the Laplace equation:

(9) $$\nabla \cdot \left(\left(\sigma +j\omega \varepsilon \right)\tilde{E}\right)=0\Rightarrow \nabla^{2}\tilde{\phi }=0.$$

We obtain the distribution of spatial electric field by solving Equation (9) subjected to induced double-layer charging condition Equation (1) on the surface of liquid metal, fixed potential phasor $\tilde{\phi }=A$ (**A** equals the specific voltage amplitude or zero for grounded state) at the electrode surface, and zero normal component of total currents $n\cdot \nabla \tilde{\phi }=0$ at insulating channel walls.

Once the distribution of potential phasor in sinusoidal steady state $\tilde{\phi }$ is known, the transient double-layer voltage drop ΔV(t)=−q0/C+Re((ϕ˜−ϕ˜drop)ejωt) can be directly calculated, in addition to the resulting voltage-dependent surface tension $\gamma =\gamma_{0}-\frac{1}{2}C{\left(\Delta V\right)}^{2}$.

#### 2.3.2. Hydrodynamics

The transient Navier–Stokes equation for laminar flow is solved in the absence of external volume force:

(10a) ∇⋅u=0,

(10b) $$\rho \frac{\partial u}{\partial t}+\rho \left(u\cdot \nabla \right)u=\nabla \cdot \left[-pI+\mu \left(\nabla u+{\left(\nabla u\right)}^{T}\right)\right].$$

Equation (10) is subjected to zero normal hydrodynamic stress at both channel inlet and outlet, since we focus on the flow behavior caused by the metal droplet itself. At the same time, zero shear stress is imposed on the air/medium interface surrounding the droplet, and a no slip wall boundary condition is applied to all other insulating channel walls. Most importantly, the mutual coupling between the electrostatics and hydrostatics embodies in the B.C. at the droplet surface that the viscous shear stress at the liquid/liquid interface is given by Equation (4) (a kind of Marangoni chaotic advection effect induced by time-varying electric field), where the time-dependent and inhomogeneous surface tension is a function of the transient double-layer voltage drop (Equation (2) and (3)), as acquired from the solution of electric field (Equation (9)).

### 2.4. Sample Preparation and System Setup

The NaOH electrolyte solutions were prepared using deionized (DI) water as the solvent and the ion concentration was adjusted ranging from 0.1 mol/L to 0.5 mol/L. The fluorescent microparticles were added into the suspending electrolyte and the high speed camera was used to capture the particle trajectories before and after being subjected to the external electric field, as represented by Figure 2c,d, respectively. The trajectories’ experiment with fluorescent microparticles (Molecular Probes, Eugene, OR USA) with 500 nm in diameter contained in NaOH aqueous solution was conducted to observe the flow pattern.

To visualize the mixing process effect, we add fluorescein (Kermel, Tianjin, China) as fluorescent dye into aqueous solution as one of the inlet laminar streams. Two solutions with identical ion concentrations are simultaneously introduced into the Y type inlet channel by two syringe pumps (PHD 2000 Series, Harvard Apparatus, Holliston, MA, USA). The Galinstan liquid metal droplet is placed into the cylindrical chamber of the open-top PDMS channel, which is filled with electrolyte solutions. The alternating current electric field is provided by two strips of copper energized with a signal generator (TGA12104, TTI, Cambs, UK), which can be monitored by an oscilloscope (TDS2024, Tektronix, Beaverton, OR, USA). The transient top view images and videos of mixing performance were recorded using a fluorescent microscope (BX53, Olympus, Tokyo, Japan) equipped with a digital camera (Retiga-2000R, Qimaging, Surrey, BC, Canada). As illustrated in Figure 2e,f, the microscopic view of the mixing effect before and after the application of the electric field were recorded. We repeated all the experiments three times and the data was presented as mean ± standard error. The Image J (Version 1.44p, NIH, Bethesda, MD, USA) was utilized to process the color intensity from the captured images.

## 3. Results and Discussion

### 3.1. Investigation of Fluid Flow Due to AC Continuous Electrowetting

#### 3.1.1. Theoretical Analysis

The dependence of surface tension between the liquid metal droplet and the electrolyte on the electrical potential difference across the EDL can be illustrated with Lippman’s equation Equation (3), reaching its maximum value *γ*_0_ at *V* = 0. Meanwhile, the pressure difference between the electrolyte and the Galinstan liquid metal droplet at each hemisphere can be obtained from the Young–Laplace equation Equation (4b). The surface tension on a curved surface will affect the final pressure difference between the liquid metal droplet and the electrolyte. Therefore, the pressure inside the Galinstan liquid metal is uniform under the circumstance of no external energized electrical field. With an external non-uniform electric field exerted, the surface tension discrepancy between the two hemispheres becomes significant, causing pressure distribution imbalance and mechanical movement of the liquid metal droplet toward the anode of the electrode in DC [44]. The pressure imbalance exists continuously over the surface of the Galinstan droplet as long as the electric field is applied, which is just the principle of the continuous electrowetting. The continuous electrowetting is an electrical analog to Marangoni effect, in which the non-uniform surface tension due to the inhomogeneous double-layer voltage drop at the ideally polarizable surface induces convective vortices over the surface of the liquid metal droplet and net flow towards the direction of transient electric field or *the region of higher surface tension* (Figure 3c,d).

In current analysis, as an AC field is applied, the induced fluid motion becomes subtle, in terms of both the *unidirectional flow component for pumping* and *local convective vortex component for mixing*. The induced pump flow caused by the globe shear stress component exhibits constant oscillating behaviors (Figure 3e). According to Equation (6), the net flow $u_{\mathrm{globe}}-\chi \frac{-q_{0}E_{0}R}{\mu }\cos(\omega t)$ oscillates *synchronously* with the background AC field at low frequencies (typically less than 100 Hz), due to complete charging of the induced double-layer (Equation (1)). With frequency approaching the reciprocal RC relaxation time $f_{RC}=\frac{\sigma \lambda_{d}}{2\pi \varepsilon R}\approx 500\;Hz$, the double-layer undergoes incomplete charging, that is, the polarity of electric field changes so fast, leaving no sufficient time for free charges to accumulate effectively with the diffuse double-layer. The double layer voltage Re((ϕ˜−ϕ˜drop)ejωt)=Ccos(ωt+θ) at such high frequencies has a magnitude *C* smaller than $E_{0}x$ induced by a low field frequency, and possesses a phase shift *θ* with respect to the background field $E_{0}\cos(\omega t)$, implying the occurrence of out-of-phase charging. As a result, due to double-layer relaxation, the surface tension gradient and the resulting fluid flow diminish as well with increasing frequency (Figure 3e). In addition, out-of-phase charging around the RC frequency makes the oscillating motion of pumping fluid flow being *asynchronous* with the applied AC electric field (results not shown). The amplitude of oscillation for the pump low component is linearly proportional to the applied voltage at a given electrode separation or the field intensity, $\left|u_{globe}\right|\propto \chi \frac{q_{0}E_{0}R}{\mu }$, due to a balance between *τ_globe_* and intrinsic viscous retardation of fluid, as has been recently utilized for unidirectional pumping of electrolyte by Tang et al. along the direction of electric field with a steady DC bias [44].

However, for achieving better mixing performance of incoming analytes, we deliberately enhance the applied voltage with the nondimensional parameter α∼O(10), so that the convective flow Equation (6d) induced by $\tau_{local}=-E_{0}^{2}Cx\cos^{2}\left(\omega t\right)$ dominates over the oscillating pump flow Equation (6c) caused by the pump stress component $\tau_{globe}=-qE_{0}\cos\left(\omega t\right)$. The most evident benefit we acquired at relatively large applied voltage is that the total convective flow |u| is mainly determined by the magnitude of local transient convection $\left|u\right|\approx \left|u_{local}\right|∼\frac{E_{0}^{2}RCx}{\mu }$, which scales with the squared value of electric field supplied. As shown in Figure 3f, with voltage amplitude ascending from 0.1 V (α ~ 1, black curve) to 2.5 V (α ~ 2.5, red curve) and increasing by twenty-five fold, the total convective flow velocity due to field-induced non-uniform surface tension increases sharply by more than two orders of magnitude, implying a quadratic voltage-dependence growth trend of the strength of local electrokinetic vortex utilized for analyte mixing.

With increasing field frequency at fixed 2.5 V, the convective and dipolar vortex flow (Figure 3b) decays due to double-layer charge relaxation (Figure 3f, red, blue and purple curves). As a result, in the absence of sufficiently strong electrical Marangoni convection at such high frequencies, mixing is mainly due to diffusion of analytes at the interface of concentration gradient, resulting in a low mixing performance. Furthermore, at large voltages, simulation results (Figure 3f) indicate that the total flow velocity has a non-zero time-averaged value, in qualitative accordance with Equation (6d).

Accordingly, *at small voltages* (α < 1 or *V*_0_ < 1 V), zero time-averaged flow induced by the pump stress at the drop surface produces *negligibly small mixing effects*. In stark contrast, *excellent mixing performance* is achievable once *the voltage is large enough* (α > 1 or *V*_0_ > 1 V) as the local Marangoni convection vortex $u_{local}∼\frac{-E_{0}^{2}RCx}{\mu }\left(\frac{1+\cos\left(2\omega t\right)}{2}\right)$ dominates over the oscillating pump component $u_{globe}∼\chi \frac{-q_{0}E_{0}R}{\mu }\cos\left(\omega t\right)$ due to the action of both *a non-zero time-averaged flow velocity* and *E*^2^ *scaling law.*

#### 3.1.2. Experimental Validation

The contact angle of the three phase contact line (TCL) is approximately 45°, and a spherical shape can be even maintained in the presence of the background electric field. The oscillation originates from a periodical reversal of the pump flow component, but not the variation of transient contact angle or standing waves induced, which is correctly captured by our physical model.

To visualize the unidirectional pump flow effect *u_globe_* and convective rolls mainly from *u_local_* in the vicinity of Galinstan liquid metal droplet under the influence of AC electric flied, fluorescent microparticles were added to the aqueous solution without inlet flow component. The trajectory of fluorescent microparticles was acquired.

At 50 Hz, according to experimental results, the liquid metal droplet located in the cylindrical chamber exhibits constant oscillating motion in phase with the AC electric field due to the effect of global stress, demonstrating the existence of oscillating pump flow *u_globe_*. In contrast, convective vortex around the droplet does not reverse direction and has a non-zero time-averaged flow velocity, validating the existence of stable electro-convection *u_local_* (see Supplementary Materials Video S1).

Finally, the total Marangoni flow enhances with increasing field intensity at 50 Hz, and becomes much quicker for relatively large voltages (see Supplementary Materials Videos S2 and S3), since the quadratic growth trend of *u_local_* dominates over the linear trait of *u_globe_* as *V*_0_ > 1 V, accounting for the domination of local Marangoni convective effect *u_local_* at large voltages, which has great potential for mixing analytes.

Approaching the reciprocal RC relaxation time *f_RC_* = 500 Hz, the convective flow diminishes with frequency, since the non-uniformity of surface tension, which gives rise to interfacial hydrodynamic shear stress, depends on the inhomogeneous induced zeta-potential within the diffuse double-layer (decaying dramatically above *f_RC_*) at the droplet surface.

Consequently, the flow behaviors of oscillating *u_globe_* along the horizontal direction and stable *u_local_* in vortex form are distinguished successfully in our experimental observations, which are in good accordance with the theoretical prediction in Figure 3.

### 3.2. Mixing Experiment

In this section, we take advantage of the dominating local electrokinetic vortex at large voltages below double-layer relaxation frequency to achieve efficient mixing of parallel incoming analytes (see Supplementary Materials Video S4). To quantitatively evaluate the mixing performance, we used the following formula to calculate the mass fraction distribution of certain cross sections perpendicular to the flow direction [17]:

(11) $$\sigma_{Me}=[1-\sqrt{\frac{1}{N}\sum_{i=1}^{N}\left(\frac{I_{i}-I_{\min}}{I_{\max}-I_{\min}}\right)}]\times 100\%,$$

where *N* is the total number of pixels, *I_i_* is the intensity at pixel *i* , while *I_max_*, *I_min_* are the intensity at pixel *i* for no mixing and complete mixing, respectively. The *σ_Me_* is 0 for no mixing or diffusion while 100% for perfectly mixing.

**Figure 4:**
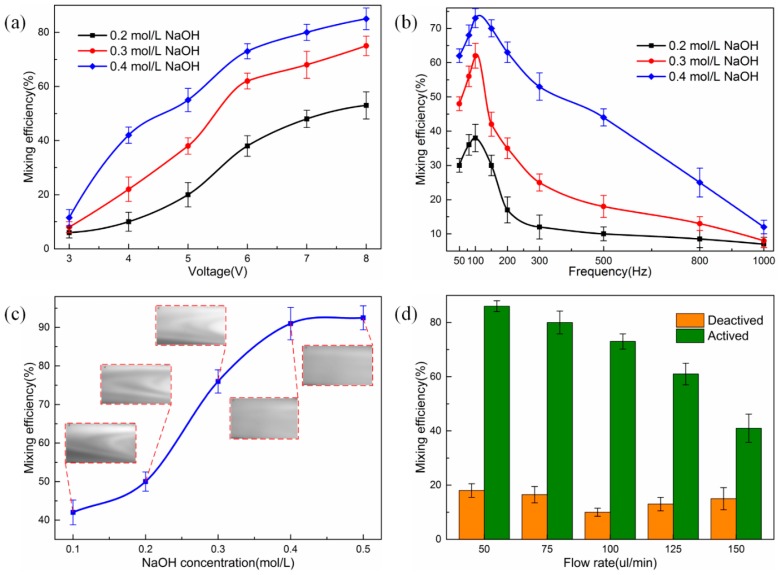
Mixing efficiency with respect to sinusoidal signals with different magnitudes and frequencies, NaOH solutions with different concentrations, as well as different inlet flow rates. (**a**) Plot for the mixing efficiency vs. voltage magnitude of the signal in a 0.2 mol/L, 0.3 mol/L, and 0.4 mol/L NaOH solution when a 100 Hz signal is applied; (**b**) plot for the mixing efficiency under different frequencies in a 0.2 mol/L, 0.3 mol/L, and 0.4 mol/L NaOH solution when a 6 Vpp signal is applied; (**c**) plot for the mixing efficiency vs. NaOH concentration after applying the electric field of 9 Vpp at 100 Hz; (**d**) mixing efficiency vs. flow rate plot, obtained when a 100 Hz, 6 Vpp sinusoidal signal is applied and the concentration of NaOH is 0.4 mol/L.

In order to evaluate the mixing performance of the high throughput mixer, we performed a series of experiments with varying frequency, magnitude of the applied voltage signals, as well as different ion concentrations of the aqueous solution. Firstly, at a constant frequency of 100 Hz, the amplitude of the signal ranging from 1 Vpp to 8 Vpp is investigated. Figure 4a presents the mixing efficiency with respect to the signal of different voltage magnitudes. Our observations demonstrate mild mixing in the absence of applied AC voltage. The mixing efficiency attains approximately 10%. This phenomenon might be accountable by a passive mixing mechanism where diffusion arising from the liquid metal droplet itself and the channel structure plays a role. Switching on the external electric field, no obvious chaotic advection can be produced unless the voltage amplitude attains 3 Vpp, in accordance with the previous conclusion at α < 1, where $\tau_{globe}\propto E_{0}$ dominates the transverse electrokinetic flow behavior. Upon increasing the voltage magnitude beyond 3 Vpp, the liquid metal droplet is actively actuated and the surface tension gradient is large enough to create convective flow eddies around the droplet surface, so $\tau_{local}\propto {E_{0}}^{2}$ dominates producing non-zero time-averaged chaotic advection in the transverse direction. Higher mixing performance can be obtained with increasing signal amplitude due to the enhanced transverse convection. The reason to enable its occurrence is that the transverse convection flow velocity $u_{local}∼\frac{-E_{0}^{2}RCx}{\mu }\left(\frac{1+\cos\left(2\omega t\right)}{2}\right)$ at large electric field is proportional to the squared value of applied voltage and the surface pressure difference between the left and right hemisphere of the droplet is intensified with the increase of surface tension discrepancy in light of the mechanism of AC continuous electrowetting. However, undesirable bubble generation occurs on the surface of the liquid metal droplet due to the electrolysis of the aqueous solution when the magnitude of the applied signal is larger than 10 Vpp at 100 Hz. We also carried out experiments on alkali concentration of 0.2 mol/L, 0.3 mol/L and 0.4 mol/L on the mixing performance under the same operating condition. We find that the higher the ionic concentration of the alkali solution, the better the mixing performance, whereas the mixing performance has no obvious changes when the ion concentration is beyond a marginal value. The reason for that will be explained in the following section. At a fixed field frequency of 100 Hz and voltage amplitude of 8 Vpp, the mixing efficiency increases from 45% to 83% with the electrolyte ion concentration increasing from 0.2 mol/L to 0.4 mol/L.

The frequency-dependence of mixing efficiency is further discussed by applying signals with changing input frequencies from 50 Hz to 1 kHz when the magnitude of the signal is 6 Vpp. As indicated in Figure 4b, the mixing efficiency enhances gradually along with the increasing frequency and reaches the maximum when the frequency is approximately 100 Hz. Nevertheless, the frequency at the best mixing is slightly different for aqueous solution with varying ion concentrations [45]. After that peak value, the mixing efficiency decreases remarkably as the AC frequency increases. There is no obvious chaotic advection when the frequency of AC signal is over 1 kHz. The reason behind the frequency-dependent mixing performance is accountable by the conductivity-dependent electrode polarization frequency $f_{electrode}=\frac{\sigma \lambda_{d}}{2\pi \varepsilon L}$ with *L* being the electrode separation, which has been well established in the field of electrochemical transport. At frequency lower than *f_electrode_*, most of the applied voltage drops across the diffuse double-layer on the electrode surface, leaving no electric field inside the liquid bulk, thereby there is no possibility for occurrence of field-induced surface tension inhomogeneity. At frequencies higher than the polarization frequency of the droplet/medium interface $f_{RC}=\frac{\sigma \lambda_{d}}{2\pi \varepsilon R}$, double-layer relaxation of the liquid metal renders again the diffuse-layer voltage drop negligibly small. As a consequence, the effective mixing can only take place within the frequency range $\frac{\sigma \lambda_{d}}{2\pi \varepsilon L}\leq f\leq \frac{\sigma \lambda_{d}}{2\pi \varepsilon R}$ (note that the optimum frequencies extend with electrical conductivity theoretically) due to electrochemical ion relaxation, in qualitative accordance with the frequency-dependent variation trend in Figure 4b. Therefore, for electrolytes with different ion concentrations, the critical frequency for no apparent mixing differs slightly and becomes larger for higher conductivities in the mixing experiment. For example, the experimental observation shows that no obvious chaotic advection occurs when the frequency is 5 kHz (1 kHz) for ion concentration of 0.5 mol/L (0.4 mol/L), which agrees well with the theory of electrochemical ion relaxation in the AC electric field.

We also investigated the mixing efficiency in response to the ion concentration of the NaOH solution, ranging from 0.1 to 0.5 mol/L. As presented in Figure 4c, the mixing efficiency is enhanced with the increment of ion concentration until reaching a plateau at *c*_NaOH_ = 0.4 mol/L and the corresponding value is 91% when a 100 Hz and 9 Vpp voltage signal is applied. We take into account that the double-layer voltage drop on the droplet surface is composed of two parts, one is the static unipolar diffuse screening cloud $\rho_{0}=-q_{0}/\lambda_{d}e^{-r/\lambda_{d}}$ caused by the fixed surface free charge at the droplet surface when no electric field acting, and the other is the induced bipolar screening cloud due to induced surface free charge on the ideally polarizable surface of liquid metal in a background electric field. Two requirements must be satisfied simultaneously to accomplish the voltage-dependent non-uniform surface tension, thereby resulting in the phenomenon of AC continuous electrowetting. The initially fixed surface free charge *q*_0_ increases with electrolyte conductivity (originates from a kind of ion adsorption mechanism), which, in turn, tends to cause a higher pressure imbalance between the two hemisphere of the Galinstan droplet, as predicted by the Lippmann equation and Young–Laplace equation, resulting in an enhancement of the electrokinetic flow velocity $u_{globe}∼\chi \frac{-q_{0}E_{0}R}{\mu }\cos\left(\omega t\right)$, which tends to improve the mixing performance by actuating transverse convective flow in a perpendicular orientation to the incoming laminar flow. However, the initial charge *q*_0_ presumably becomes saturated when the ion concentration of the electrolyte solution is up to 0.4 mol/L, beyond which the surface tension gradient $\nabla \gamma ∼q_{0}E_{0}$ is no longer able to make a change with respect to the ion concentration.

To explore the effect of inlet flow rate on mixing efficiency, we implemented experiments at varying flow rates of 50, 75, 100, 125 and 150 uL/min. Figure 4d exhibited the dependence of mixing efficiency on the flow rate without and with the driving voltage of 6 Vpp and 100 Hz, respectively. It is found that there exists slight self-mixing when the flow rate is below 75 uL/min and above 125 uL/min in the absence of electric field. This may account for the convective diffusion and oscillating impact on the droplet at low and high flow rates, respectively. The experimental observation indicated that the mixing efficiency declined with increasing inlet flow rate with the electrode pair being active. The corresponding mixing efficiency reduced from about 85% to 42% as the flow rate increased from 50 to 150 uL/min. Enhancing the inlet flow velocity reduces equivalently the time for the transverse electro-convective vortex to act on the non-uniform incoming analytes, resulting in a decrement of mixing performance. An effective solution may be an increase in the background field intensity to render the magnitude of transverse electrokinetic flow $\left|u_{local}\right|∼\frac{-E_{0}^{2}RCx}{\mu }$ commensurate with the enlarged inlet flow rate.

## 4. Conclusions

In summary, we demonstrated high throughput mixer design exploiting the AC continuous electrowetting effect of Galinstan liquid metal droplet suspended in aqueous solution. The electric field induces non-uniform and time-varying surface tension at the droplet/medium interface, which, in turn, gives rise to Marangoni chaotic advection in the vicinity of droplet consisting of unidirectional flow components for pumping fluids and local convective flow components for mixing enhancement, both of which exhibit double-layer dispersion but possess different voltage-dependent growth trends. Dominance of the dipolar flow pattern of local electrokinetic flow was utilized to realize mixing when the microfluidic device was energized with an AC signal. The mixing performance can be flexibly adjusted by tuning the voltage amplitude, field frequency as well as the ion concentration of the electrolyte solution, due to the combined effect of electrochemical ion relaxation, conductivity-dependent saturation of interfacial ion adsorption and quadratic voltage-dependent growth trend of transverse mixing flow at larger field strength. We found that the mixing efficiency can reach up to 91% in NaOH with ion concentration of 0.4 mol/L at a voltage signal of 9 Vpp at 100 Hz when the inlet flow rates is 100 uL/min. The rapid, high throughput liquid metal droplet actuator proposed herein offers the characteristic of simple manufacturing and high controllability, which potentially extends its applications in thermal transport management, microelectromechanical systems (MEMS) actuation and especially the droplet-based microdevices.

## Supplementary Materials

The following are available online at www.mdpi.com/2072-666X/8/4/119/S1, Video S1: Oscillating motion of the liquid metal droplet and the fluid flow around the droplet with the voltage signal of 5 Vpp and 50 Hz; Video S2: Fluid flow induced by local Marangoni flow with voltage signal of 0.2 Vpp and 50 Hz; Video S3: Fluid flow induced by amplified Marangoni flow with voltage signal of 5 Vpp and 50 Hz; Video S4: Fluid mixing induced by amplified Marangoni chaotic advection with voltage signal of 8 Vpp and 50 Hz.
